# The Current Case of Quinolones: Synthetic Approaches and Antibacterial Activity

**DOI:** 10.3390/molecules21040268

**Published:** 2016-03-28

**Authors:** Abdul Naeem, Syed Lal Badshah, Mairman Muska, Nasir Ahmad, Khalid Khan

**Affiliations:** 1 National Center of Excellence in Physical Chemistry, University of Peshawar, Peshawar, Khyber Pukhtoonkhwa 25120, Pakistan; naeeem64@yahoo.com (A.N.); mmuskachem@yahoo.com (M.M.); 2 Department of Chemistry, Islamia College University Peshawar, Peshawar, Khyber Pukhtoonkhwa 25120, Pakistan; nasirshah121@yahoo.com (N.A.); drkhalidchem@yahoo.com (K.K.)

**Keywords:** quinolones, analogues, gyrase, topoisomerase IV, resistance

## Abstract

Quinolones are broad-spectrum synthetic antibacterial drugs first obtained during the synthesis of chloroquine. Nalidixic acid, the prototype of quinolones, first became available for clinical consumption in 1962 and was used mainly for urinary tract infections caused by *Escherichia coli* and other pathogenic Gram-negative bacteria. Recently, significant work has been carried out to synthesize novel quinolone analogues with enhanced activity and potential usage for the treatment of different bacterial diseases. These novel analogues are made by substitution at different sites—the variation at the C-6 and C-8 positions gives more effective drugs. Substitution of a fluorine atom at the C-6 position produces fluroquinolones, which account for a large proportion of the quinolones in clinical use. Among others, substitution of piperazine or methylpiperazine, pyrrolidinyl and piperidinyl rings also yields effective analogues. A total of twenty six analogues are reported in this review. The targets of quinolones are two bacterial enzymes of the class II topoisomerase family, namely gyrase and topoisomerase IV. Quinolones increase the concentration of drug-enzyme-DNA cleavage complexes and convert them into cellular toxins; as a result they are bactericidal. High bioavailability, relative low toxicity and favorable pharmacokinetics have resulted in the clinical success of fluoroquinolones and quinolones. Due to these superior properties, quinolones have been extensively utilized and this increased usage has resulted in some quinolone-resistant bacterial strains. Bacteria become resistant to quinolones by three mechanisms: (1) mutation in the target site (gyrase and/or topoisomerase IV) of quinolones; (2) plasmid-mediated resistance; and (3) chromosome-mediated quinolone resistance. In plasmid-mediated resistance, the efflux of quinolones is increased along with a decrease in the interaction of the drug with gyrase (topoisomerase IV). In the case of chromosome-mediated quinolone resistance, there is a decrease in the influx of the drug into the cell.

## 1. Introduction

In the early 20th century, infectious diseases were the most common cause of human illness leading to death, out of which bacterial infections accounted for about one-third of these infections [1]. In addition to bacterial infections, malaria also has an enormous impact on global human health, with over 200 million cases of malaria and over 600,000 deaths each year [2]. While different therapies were used for the treatment of these diseases, the introduction of antibiotic agents opened new avenues for the treatment of bacterial infections. The invention of antibiotics such as sulfonamide, and afterward penicillin by Alexander Fleming in 1928 and synthetic antibiotics such as quinolones were some of the great milestones of science [3,4]. However, with progress in the development of antibiotics has come increasingly widespread antibiotic-resistant pathogens, which represent an unprecedented health issue [5].

**Figure 1:**
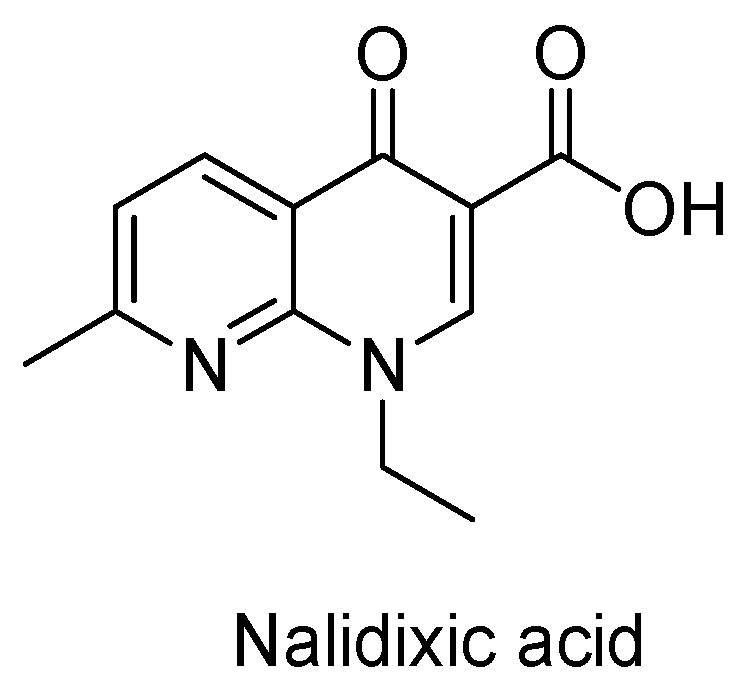
Chemical structure of nalidixic acid.

Here, we focus on an important class of synthetic antibiotics known as quinolones, that constitute an important class of biologically-active broad-spectrum antibacterial drugs [6]. The spectacular discovery of nalidixic acid, which is chemically a naphthyridone and the prototype compound of the quinolones as a byproduct in chloroquine synthesis (Figure 1) by Lesher and coworkers in 1962 initiated the improvement of quinolones [7,8]. It is present in a number of biologically-active natural products. Because of its valuable medicinal activities, quinolones have attracted a lot of attention in the field of medicinal and synthetic chemistry [9]. In the later 1960s, nalidixic acid came into clinical use for the cure of urinary tract infections caused by enteric bacteria [10]. Around the 1970s, several new generations of quinolones, with oxolinic acid being the most prominent, had been synthesized and became available for clinical use [10,11,12]. Until 1980, quinolones were a less-used class of antibacterial agents and later a second generation of quinolones was developed. In the quest for potent quinolones, it was discovered that alteration with different groups at positions C-6 and C-8 gives more effective antibacterial analogues [13]. The most significant changes in the basic skeleton of quinolones are the substitution of fluorine at C-6 and a key ring substituent piperazine or methylpiperazine, pyrrolidinyl and piperidinyl. Due to the insertion of fluorine at C-6, most clinically used quinolones are fluoroquinolones (FQ) [14,15,16].

Fluoroquinolones (formally called simply quinolones) are characterized as broad-spectrum antibacterial drugs active against both Gram positive and Gram negative bacteria. Besides broad-spectrum activity, the success of fluoroquinolones can be attributed to their properties such as good bioavailability after oral administration, relatively low toxicity and favorable pharmacokinetics [17]. Although adverse events still happen while utilizing fluoroquinolones, these are still some of the major antimicrobial agents and a lot of work has gone into the structural evolution in the framework of fluoroquinolones to make novel analogues with improved potency. Fluoroquinolones have been primarily utilized for the treatment of urinary tract infections (UTI), respiratory tract infections, sexually transmitted diseases, gastrointestinal and abdominal infections, skin and soft tissue infections and infections of bone and joints [11,18]. Besides these functions, fluoroquinolones with broad spectrum activity have been widely utilized in veterinary medicine to treat bacterial infections in food-producing animals, aquaculture and pets. Many fluoroquinolones are available on the market; however the use of these antibacterial agents highly depends on the animal species and their geographical distribution [18]. They have a wide range of medical application in livestock, poultry, fish and domestic animal in the treatment and prevention of respiratory, enteric and complex urinary tract infections [19]. Some quinolones such as 6-chloro-7-methoxy-4(1*H*)-quinolones have good antimalarial activities, with good activity against multiple stages of *Plasmodium* infection [2]. Antimalarial activity was also shown in (1*H*)-quinolone, and its tetrahydroacridine analogue, with substitution of various benzenoid ring features and aryl moieties [20].

## 2. Recent Developments in the Synthesis of Quinolones

Quinolones are very important in medicinal chemistry as they have broad spectrum antibacterial activity, and they acts as anticancer and antimalarial agents. A lot of work has been carried out on the synthesis of quinolones to develop more effective and economical analogues. A number of different methods are reported for the synthesis of quinolones. In general these, procedures involve the utilization of a preactivated substrate, a multistep strategy and harsh reaction conditions, which limit the compatibility and range of the functional groups involved in these reactions [9]. The use of transition metal catalysts has also been utilized for the synthesis of quinolones and their analogues. The catalytic activation of the C-H bond allows the synthesis of a remarkable variety of heterocyclic compounds, including quinolones [21]. The following are some of the latest reported data on the development of new methods for the synthesis of quinolones and their derivatives.

**Scheme 1:**
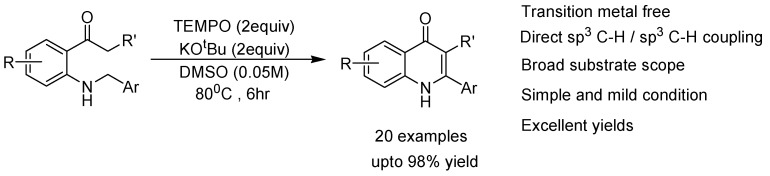
Metal-free oxidative intermolecular Mannich reaction for the synthesis of 2-arylquinone-4(1*H*)-ones from secondary amines and ketones [6].

Hu *et al.* developed a novel method in which a broad range of 2-arylquinoline-4(1*H*)-ones were synthesized by a metal-free oxidative intermolecular Mannich reaction between secondary amines and unmodified ketones (Scheme 1). The advantage of this method is that it does not require the use of any transition metal catalyst; and it occurs under mild conditions. This method can produce a number of analogues in very high yield [6].

**Scheme 2:**
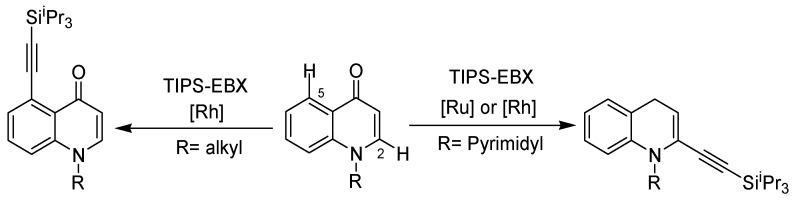
Site selective C-H alkynylation of Quinolones [22].

Kang and Hong performed alkynylation of 4-quinolones at three different positions for making quinolones analogues. In the first method, they utilized TIPS-EBX (an alkylating agent) to develop an efficient and site-selective C5 alkynylation of 4-quinolones (Scheme 2). In the second strategy a Ru(II) catalyzed C2 selective alkynylation was achieved through N-pyrimidyl group-directed cross-couplings to obtained useful C2-alkynylated 4-quinolones, while isoquinolones were developed through C3 alkynylation. These three different routes of producing quinolones analogues are site selective methods and useful for obtaining effective derivatives [22].

**Scheme 3:**
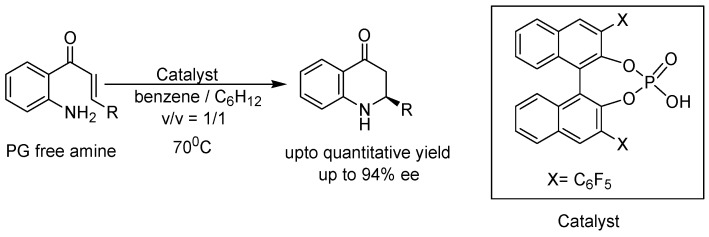
Preparation of chiral 2-substituted 2,3-dihydro-4-quinolones through chiral phosphoric acid catalyzed intra-molecular aza-Michael addition reaction.

Satio *et al.* synthesized chiral substituted 2,3-dihydro-4-quinolones in high yields using an aza-Michael addition reaction (Scheme 3). The advantage of this method is the enantioselective production of compounds and some of the analogues can be purified in a single recrystallization step. The chiral 2,3-dihydro-4-quinolones can be used for the preparation of other useful quinolone analogues. The 2-substituted 2,3-dihydro-4-quinolones halt mitosis and are useful antitumor agents [23].

**Scheme 4:**
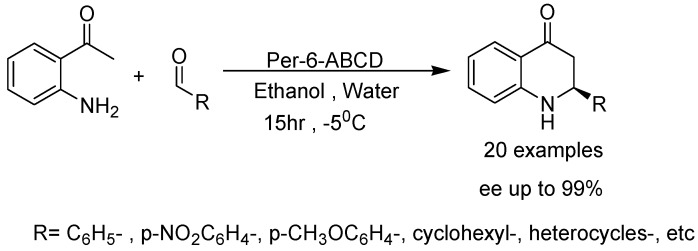
Per-6-ABCD catalyzed asymmetric one-pot synthesis of 2-aryl-2,3-dihydro-4-quinolones using substituted aldehydes [24].

Kuppusamy and Kasi synthesized enantiomerically enriched 2-aryl-2,3-dihydroquinolin-4(1*H*)-ones (in up to 99% yield) in a one pot synthesis using per-6-ABCD (Scheme 4). This synthesis method is very useful for obtaining enantiomer analogues of quinolones. The yield is very high and it is one-pot synthesis which occurs in a single step reaction with reuse of the expensive catalyst after separation from the product. The disadvantage of this method is that the low temperature and long reaction time reaction conditions are a bit cumbersome [24].

**Scheme 5:**
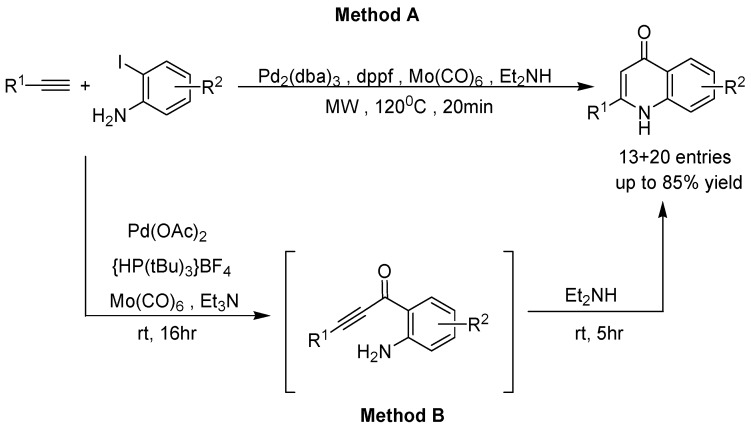
Synthesis of 4-quinolones via palladium catalyzed carbonylative Sonogashira cross-coupling reaction [25].

Ǻkerbladh *et al.* synthesized functionalized 4-quinolones from 2-iodoanilines and alkynes using two different protocols for which molybdenum hexacarbonyl was used as a solid source of CO (Scheme 5). In one of the methods, the reaction conditions include microwave heating of 120 °C and the cyclized product was obtained in 20 min in high yield. The second procedure involves the use of sensitive substituents like nitro- and bromo-groups, but the reaction is performed at room temperature. This second method is also one-pot synthesis that occurs in two reaction steps. In both of the synthesis methods palladium is used as a catalyst [25].

**Scheme 6:**
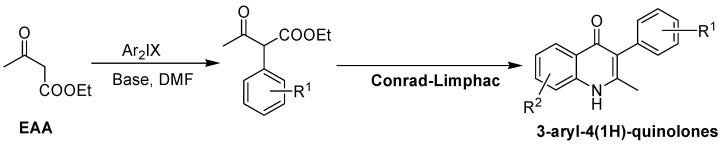
Metal free arylation of ethylacetoacetate with hypervalent diaryliodonium for the synthesis of 3-aryl-4(1*H*)-quinolones [26].

Monastyrskyi *et al.* developed a protocol for the synthesis of 3-aryl-4(1*H*)-quinolones from ethyl acetoacetate (EAA) using hypervalent diaryliodonium salts under mild and metal-free conditions (Scheme 6). This method has also been applied for the synthesis of the antimalarial compound ELQ-300. This compound is active against *Plasmodium falciparum* and *Plasmodium vivax* at all stages of its lifecycle and is in the preclinical testing stage. The toxicity of this product is not yet reported, but the preclinical data provides important information concerning efficacy. The usefulness of this synthetic method is its product that is highly desirable, as malaria is one of the deadliest diseases, affecting millions of people in Asia and Africa [26].

**Scheme 7:**
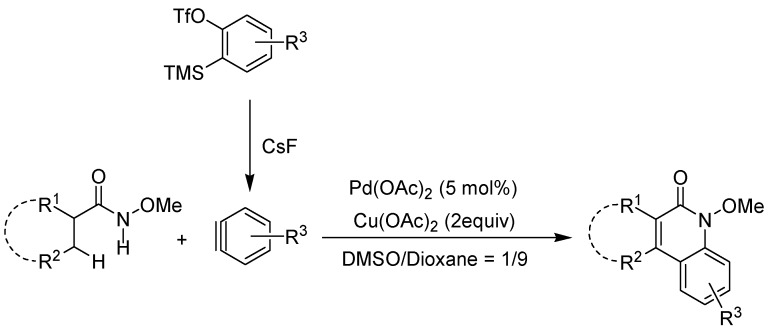
Palladium catalyzed-oxidative annulation of acrylamide with strained arynes for the synthesis of quinolones [27].

Wang *et al.* synthesized a wide variety of quinolones in a high yield in one step process using a mild N-H activation/Heck reaction method (Scheme 7). This process involves the palladium catalyzed-oxidative annulation of acrylamide with strained arynes. This technique was made possible for the first time as previous attempts by other researchers resulted in lower yields [27].

**Scheme 8:**
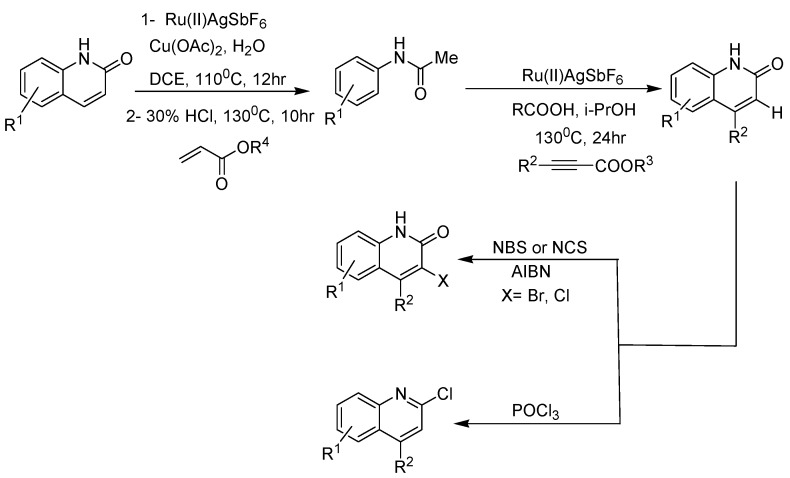
Synthesis of 2-quinolones through ruthenium catalyzed cyclization of anilides with substituted propiolates or acrylates [28].

Manikandan and Jeganmohan prepared 2-quinolones by Ru-catalyzed cyclization of anilides with propiolates or acrylates with further conversion into 3-halo-2-quinolones and 2-chloro-quinolones (Scheme 8). The 2-quinolones produced through this mechanism are very useful for the synthesis of other quinolone derivatives, and are prepared in excellent yield. The mechanisms proposed for the above reactions were strongly supported by experimental evidence and deuterium labeling studies, suggesting easy isolation of reaction intermediates during synthesis. The halogenated products made through such synthesis methods still need to be assayed for their toxicity in mouse models for their future medicinal value [28].

Suet *et al.* synthesized and evaluated bisaryl quinolones for biological activities against *P. falciparum* [29]. Pateland his coworkers synthesized a series of novel pyridoquinolone analogues by introducing sulfonamide, urea, thiourea, amine, amide, piperazine and β-lactam moieties in the pyridoquinolone molecule. These quinolones on the one hand inhibit gyrase and topoisomerase IV of bacteria while the other attached groups are also inhibitors of specific metabolic pathways. These medicinal molecules have antibacterial and antifungal activities and some of them are even active against drug resistant *Mycobacterium tuberculosis* strains. However there is a need to test these novel quinolones in mammals for their potential toxicity [30,31,32,33,34,35,36]. Komarnicka *et al.* made phosphine derivatives of sparfloxacin in high yield by treating sparfloxacin with methoxy(diphenyl)phosphine. The oxide derivative was made by treating the aminomethyl(diphenyl)phosphine analog of sparfloxacin with 35% hydrogen peroxide in chloroform at room temperature with continuous stirring for 3 h. The aminomethyl(diphenyl)phosphine analog of sparfloxacin showed antibacterial activity of similar nature to sparfloxacin, while the oxide form had high antitumor activity against cancer cell lines [37]. One of the beautiful modifications in the existing quinolone is the production of a series of 1,2,4-triazole-ciprofloxacin hybrid compounds that have potential antibacterial potency against both Gram-positive as well as Gram-negative bacteria. These 1,2,4-triazole-ciprofloxacin hybrid compounds were more active than ciprofloxacin against both vulnerable and quinolone resistant bacteria. Such promising hybrid triazole analogues of other quinolone compounds should also be prepared and tested against both resistant and non-resistant bacterial strains that cause different diseases. In addition to determine their action against pathogenic bacteria, they should also be tested for viral infections, especially HIV [38]. A number of new natural antibiotics are being discovered that can target fluoroquinolone resistant bacteria (FQR). For example deoxynymbomycins can target the mutant bacterial DNA gyrase and can be used against FQR bacteria but the chances of resistance to this new drug are still there [39]. Similarly, the FQR *Neisseria gonorrhoeae* type II topoisomerase can be inhibited by a novel compound called spiropyrimidinetrione AZD0914 [40] so on one hand we have the continuously evolving resistant strains to quinolones and other antibacterial agents, but on the other hand scientific community is working hard to design and synthesize new drugs for the resistant bacterial strains.

## 3. Analogues of Quinolones

**Figure 2:**
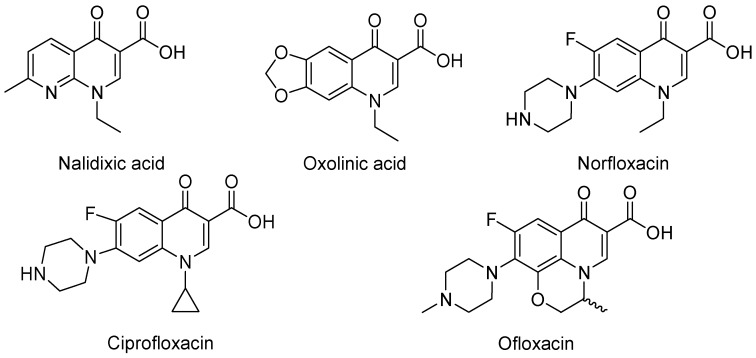

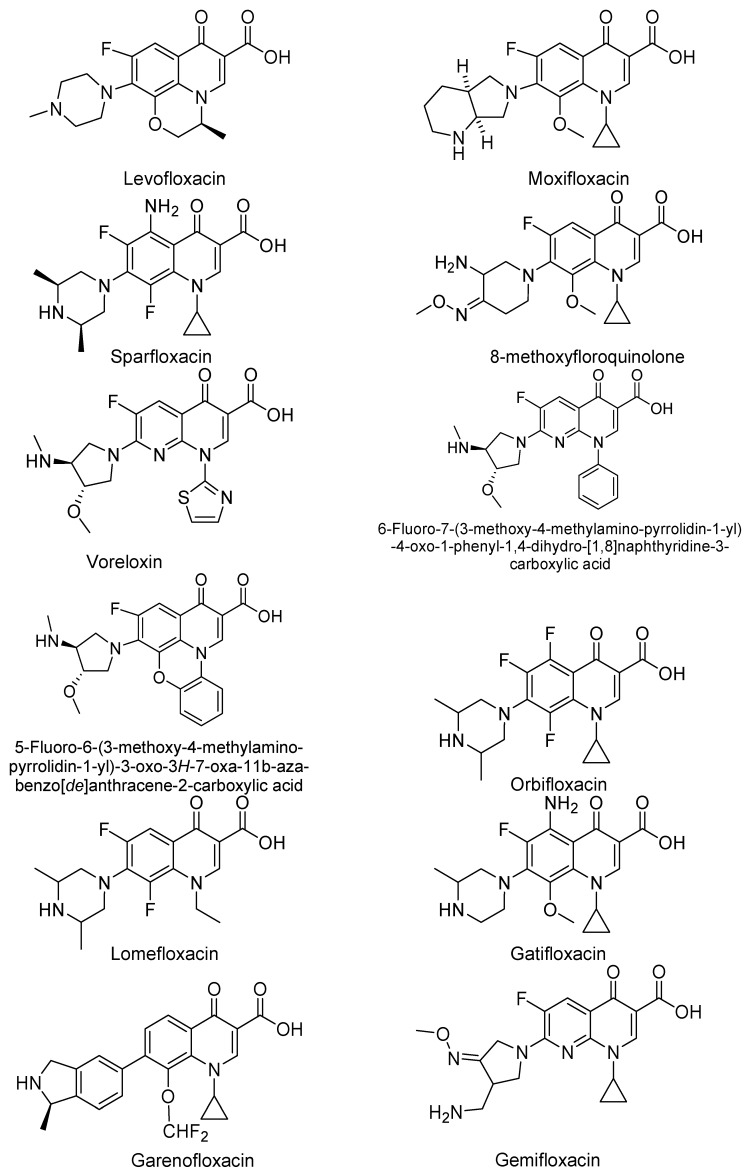

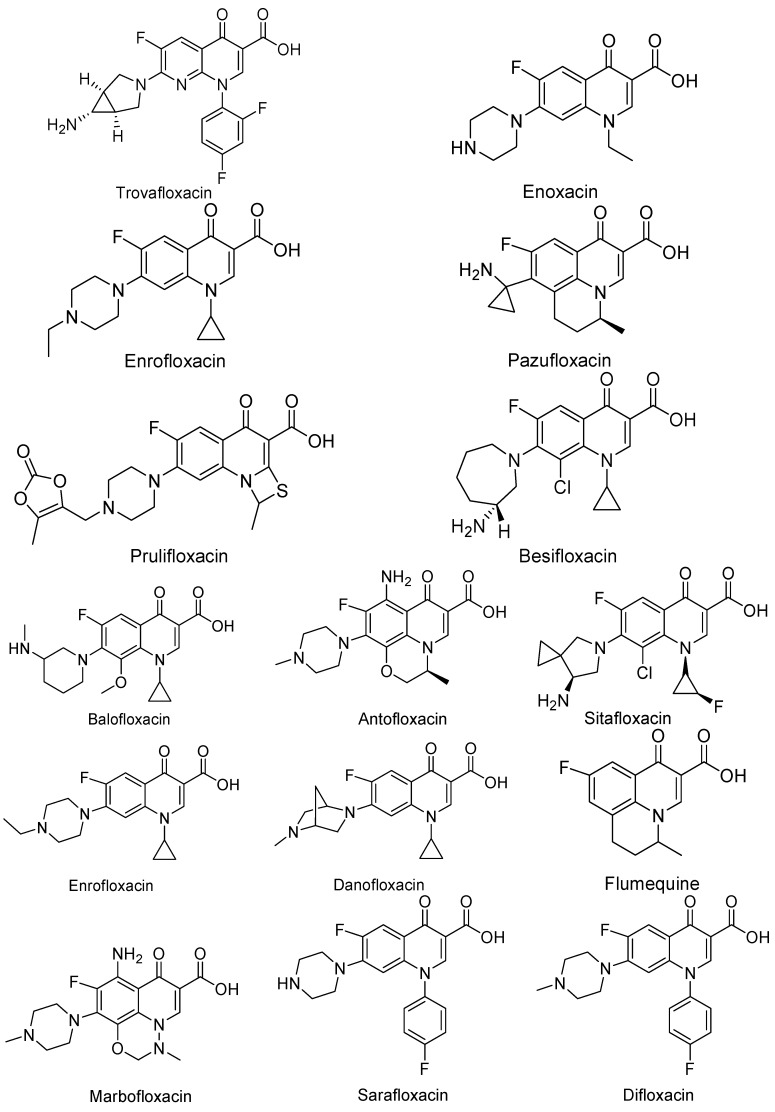
Common analogues of quinolones used for the treatment of different diseases.

The first member of the quinolone family was nalidixic acid, which was synthesized as a byproduct in the synthesis of chloroquine. Later, it was modified in order to enhance its activities. One modification was the substitution of fluorine at the C-6 position, which was found to provide good antibacterial activities. Moreover, substitution of piperazine or methyl piperazine, pyrrolidinyl and piperidinyl moieties was also determined to result in increased antibacterial activities. Following are the analogues of quinolones available in the common literature (Figure 2) [15,16,41,42,43,44,45,46,47,48].

## 4. Clinical Use

There are a number of quinolone analogues used in clinical practice all over the world due to their increased antibacterial range and favorable pharmacokinetics, while the search for new hybrid next generation quinolones is ongoing. Among the most suitable quinolones candidates that are highly prescribed are gatifloxacin, levofloxacin, ciprofloxacin. There are several quinolones that have more toxicity for the patients than usefulness, like halogenated quinolones, but such toxic analogues should be used when no other option is available and the toxic damage is less as compared to the pathogen harm [49]. The quinolones are also suitable choice for lung infections like cystic fibrosis. Clinafloxacin is cytotoxic, but it can be used against multi-drug resistant *Burkholderia cepacia* [49]. *Pseudomonas aeruginosa*-related respiratory diseases can also be treated best with anionic fluroquinolones. In *P. aeruginosa* based diseases like cystic fibrosis where they form a thick biofilm of anionic molecules such as rhamnolipids and alginate, it is difficult for most antibiotics to penetrate and inhibit bacterial growth. However the negatively charged fluoroquinolones have the ability to cross the mucus and thus have better pharmacodynamics due to their negatively charged nature [50].

Some quinolones like ciprofloxacin, levofloxacin, moxifloxacin, ofloxacin, and gatifloxacin are also approved by Federal Drug Administration (FDA) for pediatric use for different diseases like conjunctivitis, otitis, sinusitis, respiratory tract infections, pneumonia, UTI and gastrointestinal diseases [51]. Fluoroquinolones can be used to cure tuberculosis (TB) as other active agents used for treatment take six months or more, while FQ can shorter this time span [52]. The development of multi-drug resistance in *Mycobacterium tuberculosis* strains has already alarmed the medical community as these strains are highly resistant to antibiotics. Quinolones also suffer the same fate as antibiotics as several strains of *M. tuberculosis* generate mutations in their DNA gyrase enzyme encoded by GyrA and GyrB genes [53,54,55]. Mutations in the amino acids Ala90 and Asp94 of GyrA of several strains of *M. tuberculosis* resulted in high levels of resistance to levofloxacin [56]. The level of resistance to various analogues of quinolones is different in different countries [54,57,58,59,60,61].

The quinolones can also be used to treat diseases that occur as a result of excessive production of aldosterone. Some quinolone analogues can inhibit a group of mitochondrial cytochrome P450 (CYP) enzymes that are involved in aldosterone biosynthesis. Thus they can be used for the treatment of heart related diseases like hypertension, primary aldosteronism, congestive heart failure, cardiac fibrosis, ventricular remodelling, and diabetic nephropathy that occurs due to high level of aldosterone production [62].

Beside the antibacterial role of quinolone, there are certain quinolonyl diketo acid analogues that are active against human immunodeficiency virus (HIV). They act against multiple viral enzymes that include reverse transcriptase, integrase and ribonuclease. These agents bind in the active site of integrase, ribonuclease and reverse transcriptase with the help of magnesium ion with acidic residues in their catalytic site and in that way they halt the function of these enzymes [63,64,65,66,67]. These quinolonyl diketo acid analogues are synthesized by introduction of various alkylating group at the nitrogen atom of the quinolinone ring [63]. Similarly the mono and bifunctional quinolinonyl diketo acids are also potent inhibitors of strand transfer function of HIV integrase, which are synthesized by treating aniline with ethyl orthoformate and ethyl acetoacetate [65,66,67]. The antitumor activities of quinolones were also investigated by different research groups. A Mannich base can be prepared from ciprofloxacin and kojic acid that possesses antitumor activities. This Mannich base and its complex with copper are toxic to cancerous cells in different ways that include inhibition of replication, disruption of polarization of mitochondrial membrane resulting into loss of ATP activity of cancerous cell [68]. It is also reported that quinolones acts against other types of cancer where its mode of action is suggested to be inhibition of topoisomerase II.

In veterinary medicine the quinolones are also prescribed due to their favorable pharmacodynamics and pharmacokinetics, especially for various types of diseases and wounds in dogs and cats. The commonly prescribed fluoroquinolones for different dog and cat diseases includes ciprofloxacin, difloxacin, enrofloxacin, marbofloxacin, orbifloxacin and pradofloxacin [69,70].

## 5. Targets of Quinolones

The main targets of quinolones are DNA topoisomerases. These enzymes are essential for DNA replication, transcription, recombination and condensed DNA remodeling, and function by carrying out transient single- and double-strand breaks. The topoisomerases modulate the supercoiling of DNA to enable proper function and interaction with proteins. These enzymes also have a fundamental role in most nucleic acid processes, like helping to control levels of DNA under- and over-winding, as well as removing knots and tangles from bacterial chromosomes [71,72,73]. The enzymes that cleave just one strand of the DNA are called type I topoisomerases, and they are further divided into type IA, based on the protein covalent linkage to 5′-phosphate, or type IB if the linkage is to 3′ phosphate of the DNA strand. The type II topoisomerases cleave both strands of the double stranded DNA concurrently. The binding and subsequent hydrolysis of ATP with type II topoisomerase results in a conformational changes in DNA. In most of the bacteria, two different but structurally similar type II topoisomerases are present, namely: gyrase and topoisomerase-IV. Both gyrase and topoisomerase-IV are heterotetrameric subunits. The gyrase has two each GyrB and GyrA subunits while the topoisomerase-IV contains two subunits each of ParE and ParC subunits in Gram-positive bacteria. On the other hand GrlA and GrlB are the subunits of topoisomerase-IV in the case of Gram-positive species [71,74,75,76,77]. Schoeffler and Berger have reviewed the structure and function of the type II topoisomerase mechanism in great detail, as well as the role of their ATP-dependent domains [76,78,79]. The processes of ligation and DNA cleavage, which make up the central part of enzyme function, use a non-canonical two-metal ion mechanism [80,81]. Gyrase and topoisomerase-IV make staggered cuts in DNA that are four base pairs apart and on opposite strands, leaving a 5′ overhang. In this process, a covalent bond is formed between active site tyrosine residues and the newly generated 5′ DNA termini, in order to maintain a genomic integrity [16,71,74,75,77]. It is noteworthy that humans also have type II enzymes; topoisomerase IIα and topoisomerase IIβ which have significant amino acid sequence similarity to bacterial enzymes, but in humans, the genes that code for subunits A and B are fused, forming a single polypeptide chain. Thus, the human type II enzymes function as homodimers. Due to this difference, the clinically-relevant quinolones can discriminate between the human and bacterial type II topoisomerases [16,71,82].

## 6. Mode of Action of Quinolones

It was first recognized in 1977 that the bacterial enzyme gyrase is the primary target of quinolones. Later, with the discovery of topoisomerases, it was suggested that this enzyme could also be a target of quinolones. Analysis of *Escherichia coli* strains carrying drug-resistance mutations in these enzymes showed that quinolones inhibit gyrase and topoisomerase-IV as primary and secondary targets, respectively [8]. There are several high-resolution structures of topoisomerase in different catalytic modes with DNA, which have shown how the topoisomerases modify the topology of DNA [83,84,85]. In a recent investigation, the interaction of quinolones and type II topoisomerase of human and bacteria are mediated by different types of interactions. It was shown that the quinolones target the gyrase and topoisomerase-IV enzymes and inhibit bacterial growth [86]. These enzymes have the potential to fragment the cell genome, which is essential for cell survival and reproduction. Quinolones inhibit cell growth and division by increasing the concentration of enzyme-DNA cleavage complexes [16,73]. Thus quinolones are referred to as topoisomerase poisons as they convert gyrase and topoisomerase IV into cellular toxins [87].

## 7. Interaction of Quinolones with Topoisomerases

**Figure 3:**
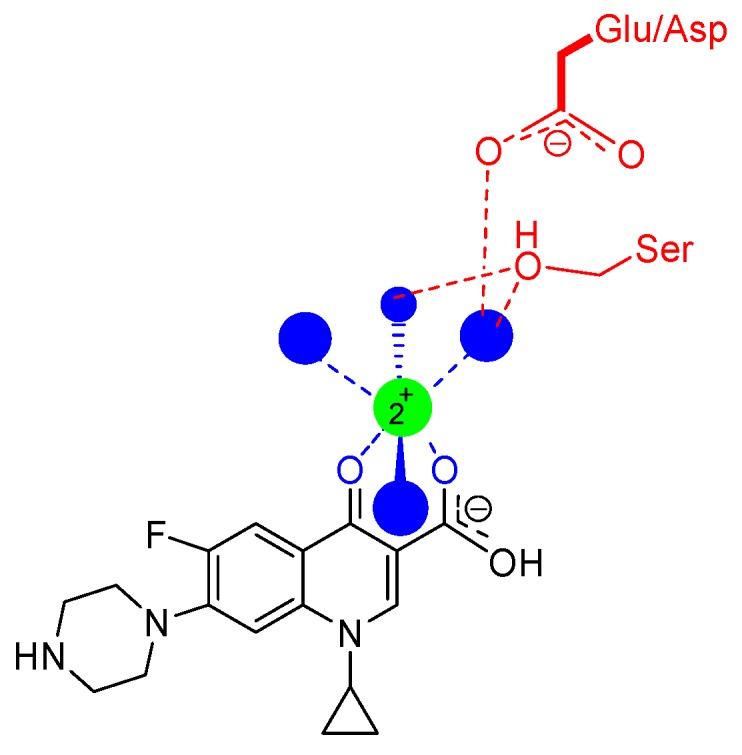
Quinolone-enzyme interaction [86].

The interaction of drugs with proteins plays an important role in stabilizing the complexes. In the case of Gram-negative bacteria, quinolones bind to gyrase, while in Gram-positive bacteria, the drugs preferentially bind to topoisomerase-IV [88,89]. The amino acids that are usually associated with quinolone-resistance are GyrA-Serine 83 and GyrA-aspartate 87 in *E. coli* [90,91,92,93,94]. Mutation of these two amino acids to alanine results in resistance to quinolones, which showed the importance of these two amino acids in drug binding [95]. Consequently, it is has been assumed that these two amino acids play a crucial role in mediating quinolone-enzyme interactions. A large number of crystallographic studies have been performed to explain the quinolone-enzyme interactions. These structures showed how the drugs interact with both the enzyme and DNA and how these drugs halt the normal process of DNA cleavage and religation. There are differences in the interaction of drugs with the enzyme among the resolved structures as they vary with the species and the drug used. However they are helpful in designing more potent analogues that can bind more tightly with the enzyme and DNA. In addition, molecular docking and simulation of such structures and different quinolone analogues will be helpful in modelling the interactions to help design new drugs [83,84,96,97,98,99,100]. Osheroff and coworkers provided most of the data about the interaction of quinolones and topoisomerases [16,45,73,77,81,82,86,101,102,103,104,105,106,107,108,109]. In a recent study, the quinolone structure was placed near the serine and acidic residues, but it was found that the amino acids were not close enough to mediate a direct binding to drugs. On the other hand, a structure of a quinolone complex was found having a non-catalytic Mg^2+^ chelated by the C3/C4 keto acid of the drugs [86]. The Mg^2+^ was in coordination with four molecules of water, two of which were located close enough to the serine and acidic residues favorable for hydrogen bonding (Figure 3). The above studies suggested that the interaction of water to metal bridged the quinolone to the enzyme [83,84,86,93,97,99]. It was also observed that a mutation in the place of serine or nearby another amino residue limits the variety of metal ions that support the drug activity [101]. Thus mutation resulted in decrease in affinity of the enzyme for quinolones at the noncatalytic Mg^2+^ site. Additionally, these mutations decrease the affinity of gyrase and topoisomerase-IV for quinolones and in the case of mutation in both residues, there is a complete loss of relevant quinolones to stabilize cleavage complexes [16,86,103].

Thus the existence of a metal ion-water complex verifies that serine and acidic residues act as anchor points that coordinate the bridge to the enzyme and that the water-metal ion bridge is the primary interaction between the drug and bacterial type II enzymes [104]. It is noteworthy that the type II topoisomerase of humans lacks serine and acidic residues to affix the water-metal ion bridge and is therefore not capable of utilizing this critical mechanism to interact with quinolones. That is why the toxicity of quinolones is also minor for humans [104]. These differences in topoisomerases structure provide a base by which quinolones differentiate between the bacterial and human topoisomerases and opens beneficial avenues for this class of drugs.

## 8. Bacterial Resistance to Quinolones

Like other antibiotics, quinolones face resistance from infectious bacteria due to their widespread use and this resistance is a major clinical issue. Bacterial resistance to quinolones is increasing and it is common reported throughout the world. Most vancomycin-resistant enterococcus (VRE) and methicillin-resistant *Staphylococcus aureus* (MRSA) are also cross-resistant to fluoroquinolones [109,110]. Bacterial resistance to quinolones can be classified into three categories [16].

## 9. Target-Mediated Quinolones Resistance

Mutation in the target site of quinolone: gyrase (GyrE subunit) and topoisomerase-IV (ParC subunit) of bacteria are the most common and they result in resistance to quinolones. The serine and acidic residue of amino acids that bind the water-metal on bridges undergo mutation frequently results in ten times less binding affinity for quinolones [95,104]. Most probably, the disturbance in the water-metal ion bridge leads to the quinolone resistance. Normally, mutations in serine comprise above 90% of the mutant pool in both clinical and laboratory isolates, while changes at the acidic residues comprising the bulk of other mutations [92,93]. In most of the resistant strains of bacteria, serine is the most common residue that is mutated: to leucine in MRSA and to isoleucine, arginine or tyrosine in GyrA subunit in VRE. After the mutation in gyrase and topoisomerase-IV, wild type DNA cleavage does not stop in the absence of drugs, however quinolones have little ability to enhance the cleavage of enzyme mediated DNA [16,102,111,112,113]. In addition, the binding capability of quinolones is largely reduced and it loses much of their ability to inhibit DNA ligation or to make enzyme-DNA-drug complex [16,104].

## 10. Plasmid-Mediated Quinolone Resistance

The second method of bacterial resistance to quinolones are plasmids that carry quinolone resistance genes that are also a rising clinical issue, commonly causing low level resistance (less than 10-fold) [114,115,116] though a 250-fold resistance has also been reported [117,118]. The transmittance of plasmids mediated quinolone resistance from one bacterial generation to another can be done horizontally via conjugation as well as vertically (target mediated resistance is transmitted only vertically). The plasmids responsible for quinolones bear additional genes which offer resistance to other drug classes [115,119,120]. Plasmid mediated quinolone resistance is due to three group of genes. These genes are qnr genes, AAC (6)-Ib-cr and the third group comprised of efflux pumps. The qnr genes offer resistance by decreasing the binding of DNA to gyrase and topoisomerase-IV; as a result there is decrease in the target site of chromosomes. The efflux pump is also a plasmid encoded protein that causes resistance to quinolones [121,122]. The second gene associated with quinolone resistance is an alternative protein of an aminoglycoside acetyltransferase having a mutation at two specific points. The enzymes decrease the activity of the quinolones by acylation of unsubstituted nitrogens of the C7 piperazine ring in norfloxacin and ciprofloxacin [123].

## 11. Chromosome-Mediated Quinolone Resistance

The concentration of quinolones inside the cell is regulated by the diffusion-mediated uptake of drugs and pump-mediated efflux. For a drug to enter into cell of Gram-negative bacteria, it must pass through extra barriers. Therefore the influx of drug into Gram-negative species is facilitated by protein channels called porins. The down regulation of porins expression leads to low level quinolone resistance [109,118,119]. Along with the plasmid encoded efflux pump, if the expression of chromosomes encoded efflux pumps are enhanced, it will also offer low level resistance. Usually the mutations in regulatory proteins cause the up-regulation of these efflux pumps [109,124]. The resistance that arises due to lowering the concentration of quinolones within the cell may not be a big clinical issue but it provides a favorite environment to other kind of resistance to antibacterial agents [124,125].

## 12. Conclusions

Quinolones are utilized worldwide as broad spectrum antibacterial agents, effective against both Gram-positive as well as Gram-negative bacteria. There are several quinolones derivatives that also work as antimalarial, antiviral and anticancer agents. After their initial synthesis as a byproduct of chloroquine, quinolones evolved as a separate class of synthetic drugs. Currently different synthetic methods are explored to synthesize more effective novel analogues of quinolones. Due to the overuse of these drugs, certain bacteria produce resistance to them and these strains of bacteria are now a common threat worldwide. Several mechanisms for resistance have been explained among which mutation at the target site is common. There is a need to explore new synthetic routes for novel quinolone analogues that are cost effective, less cytotoxic and have better pharmacokinetic and pharmacodynamic properties. This latest information about quinolones can help scientists to develop such analogues which can avoid resistance to quinolones and can extend their clinical utilization in the future.
